# Development of highly aggressive mantle cell lymphoma after sofosbuvir treatment of hepatitis C

**DOI:** 10.1038/bcj.2016.16

**Published:** 2016-03-11

**Authors:** R J Lin, T Moskovits, C S Diefenbach, K B Hymes

**Affiliations:** 1 Division of Hematology & Medical Oncology, New York University School of Medicine, Laura and Isaac Perlmutter Cancer Center, New York, NY, USA

Previous clinical epidemiological studies have demonstrated an increased risk of B-cell lymphoproliferative disorders in patients with chronic hepatitis C virus (HCV) infection.^1^ The most common B-cell non-Hodgkin's lymphoma associated with HCV is marginal zone lymphoma (MZL), including splenic and extranodal (mainly non-gastric) forms. However, associations with mucosa-associated lymphoid tissue lymphoma, lymphoplasmacytic lymphoma (LPL) and diffuse large B-cell lymphoma (DLBCL) have also been reported, although an association between HCV and mantle cell lymphoma (MCL) is uncommon.^1^ These observations were substantiated by the EPILYMPH study that identified DLBCL, MZL and LPL as most frequently associated with HCV infection.^2^ Furthermore, the large International Lymphoma Epidemiology Consortium (InterLymph) case–control study quantified the lymphoma risk associated with HCV infection as follows: DLBCL (odds ratio (OR)=2.24)), MZL (OR=2.47) and LPL (OR=2.57).^3^

Despite these findings, a causal relationship between HCV infection and lymphoma remains unclear, confounded by factors, such as the method of HCV assessment, the selection of normal controls and the classification of lymphoproliferative disorders.^4, 5^ The role of chronic and continuous viral antigenic stimulation inducing an ongoing B-cell response with subsequent genetic events leading to clonal and malignant evolution has been described,^1, 6^ and the observation of lymphoma regression after HCV treatment with interferon and ribavirin gives additional plausibility to this hypothesis.^7, 8, 9^ Here, we described the novel finding of two patients who presented with aggressive MCL in the context of clearance of chronic HCV infection.

The first patient was a 69-year-old Caucasian male Podiatrist with hypertension, and newly diagnosed HCV who had completed 12 weeks of sofosbuvir/ribavirin treatment in December 2014. One month later he presented with progressive anorexia, weight loss and lower extremity weakness. No contributory family or social history was elicited. On admission, he had diffuse lymphadenopathy, massive hepatosplenomegaly, bilateral lower extremity erythematous rash and spinal cord compression. Laboratory studies were notable for a whilte blood cells 13.7 with 35% monocytes, hemoglobin of 9.7, platelet of 386, elevated lactate dehydrogenase of 5872 and elevated uric acid of 13.3 with acute kidney injury with creatinine of 2.8. HCV viral load was undetectable and liver function tests were within normal limits. Human immunodeficiency virus/epstein-barr virus/cytomegalovirus (HIV/EBV/CMV) serology was negative. Skin biopsy showed leukocytoclastic vasculitis. Lymph node biopsy revealed a ‘double-hit' blastic MCL with cyclin D1 (t11;14) and c-Myc (t8;14) translocation. Ki-67 proliferation index exceeded 90%. He was being treated with R-hyperCVAD regimen with partial response.

The second patient was a 61-year-old Hispanic male with chronic hepatitis C and cirrhosis who had failed previous interferon treatments but completed 24 weeks of sofosbuvir/ribavirin treatment resulting in clearance of HCV viral load in July 2014. Within 1 month, he developed progressive anorexia, weight loss and night sweats along with increased inguinal lymph nodes. No contributory family or social history was elicited. Positron emission tomography/computed tomography (PET/CT) showed increased spleen size with maximal standardized uptake value of 4–6. Laboratory studies revealed a whilte blood cells of 14.6 with 64% lymphocytes, hemoglobin of 12.5, platelets of 80 with lactate dehydrogenase of 852 and creatinine of 1.0. HIV was negative as was the HCV viral load. Lymph node and bone marrow biopsy showed blastic MCL with cyclin D1 (t11;14) translocation and Tp53 mutation. Ki-67 proliferation index was between 60–80%. He could not tolerate R-hyperCVAD, and had a transient response to ibrutinib.

There are no published reports linking immediate development of blastic lymphomas after successful treatment of HCV infection. In general, the effect of HCV treatment on lymphoma is favorable.^7, 8, 9^ For example, Michot *et al.*^9^ investigated the role of antiviral therapy in 116 HCV-associated B-cell lymphoma patients (45% MZL, 45% DLBCL and 22% others) and found better overall survival in patients treated with antiviral therapy. Five MCL patients were included and no difference in outcome was reported.^9^ Finally, a case of refractory MCL resistant to chlorambucil, splenectomy, rituximab, and seven cycles of fludarabine, mitoxantrone and rituximab had a complete remission with undetectable HCV viral load within 1 month of treatment with ribivirin and interferon.^10^

Several potential explanations may account for our observation. The aggressive MCL may be purely coincidence with HCV treatment in regard to timing, or have originated from an indolent MCL whose development was accelerated by HCV infection and/or treatment. This is supported by the proposed molecular model of multistep pathogenic evolution of blastoid MCL in which the initial cycle D1 overproduction is followed by additional genetic alterations related to DNA damage repair and the cell death pathways.^11^ Alternatively, sofosbuvir, a new class of specific nucleotide analog inhibitor of HCV NS5B polymerase, may have had unwanted effect on multistep lymphomagenesis, even though it is active in treating HCV-associated MZL.^12^ Finally, restoration of host T-cell and B-cell-mediated immunity through HCV eradication may result in dysregulated host immune response facilitating additional clonal evolution of MCL analogous to the immune-reconstitution inflammatory syndrome in HIV infection,^13^ although immune-reconstitution inflammatory syndrome associated with HCV treatment has not been reported in the literature.
